# 1359. Understanding Data Completeness from Sites Participating in the N3C Limited Data Set

**DOI:** 10.1093/ofid/ofad500.1196

**Published:** 2023-11-27

**Authors:** Yuval Koren, Shivani Aggarwal, Daniel Poscover, Charles Barr

**Affiliations:** Graticule,Inc., Newton, Massachusetts; Landmark Science, Inc., Newton, Massachusetts; Graticule, Inc., Newton, Massachusetts; Graticule,Inc., Newton, Massachusetts

## Abstract

**Background:**

Numerous data sources such as registries utilize data collected across multiple sites, typically across a geographic region. Understanding the degree to which individual sites contribute core data components such as diagnoses, treatments, procedures, and measurements, is an essential step when conducting analyses using a multi-site database. The National COVID Cohort Collaborative (N3C) is the largest repository of deidentified clinical data from 18.9 million patients tested for or diagnosed with COVID-19 in the United States, and has been cited in over 150 publications, preprints, and presentations. Data is systematically collected across electronic health records from multiple sites and harmonized into the OMOP Common Data Model (CDM). Here, we aim to describe data completeness from participating sites using the N3C Level 3 Limited Data Set.

**Methods:**

Key OMOP CDM data tables (measurement, drug_exposure, condition_occurrence, and procedure_occurrence) from two N3C data releases six months apart (August 2022 and February 2023) were assessed. A site was considered to contribute to a key data table if a threshold of at least 1,000 records and lag of no more than 5 months within 6 months prior to the data release date through the data release was observed for that site. Records with dates after the data release date or with missing dates were not included. The proportion of sites with data in at least one key table and data in all key tables in the August 2022 and February 2023 data releases was determined.

**Results:**

Of the 77 sites identified in the N3C Limited Data Set in August 2022, 76.6%-83.1% had data in at least one key table and 75.3% (N=58) had data in all four key tables. A drop of 6.8% in the number of sites with data in all 4 key tables was observed in the February 2023 data release. The condition_occurrence table was most impacted by site attrition (12.5%) between these data releases, while the drug_exposure was the least impacted (3.2%).

**Conclusion:**

We observed a minor heterogeneity and a high proportion of sites contributing key data tables through the two N3C data releases under investigation. Assessing such sites characteristics during the study design phase may be an essential step when designing high-quality real-world studies that utilize multi-site data.

**Disclosures:**

**Yuval Koren, MSc**, AstraZeneca: Grant/Research Support **Daniel Poscover, MBA**, AstraZeneca: Grant/Research Support **Charles Barr, MD, MPH**, AstraZeneca: Grant/Research Support

